# Topic modeling of workplace bullying discourse following legal regulation in South Korea

**DOI:** 10.3389/fpubh.2026.1811545

**Published:** 2026-05-05

**Authors:** Hyunjoo Oh, Yeajoo Kim, Hipyo Ahn, Dong-gwi Lee

**Affiliations:** 1 Yonsei Psychological Science Innovation Institute, Yonsei University, Seoul, Republic of Korea; 2 Department of Psychology, Yonsei University, Seoul, Republic of Korea

**Keywords:** Latent Dirichlet Allocation, occupational health, online discourse, topic modeling, workplace bullying

## Abstract

**Background:**

Workplace bullying constitutes a major psychosocial hazard with downstream consequences for mental health, work performance, and population health. In South Korea, statutory provisions addressing workplace bullying were introduced under the Labor Standards Act and have been in effect since July 16, 2019. However, empirical evidence remains limited regarding how workers' self-reported, everyday experiences and response pathways are articulated and patterned in the post-legislation context, particularly within online help-seeking narratives explicitly labeled as “workplace bullying.”

**Methods:**

A total of 10,788 workplace-bullying-related question posts were collected from Naver Knowledge iN (South Korea) between July 16, 2019 and July 31, 2025. Comments and answers were excluded, and deduplication, minimum-length filtering, and relevance screening were applied. Korean text was preprocessed, and Latent Dirichlet Allocation (LDA) topic models were fitted. Topic solutions were evaluated using coherence and perplexity metrics; a 12-topic solution (K = 12) was selected for the full corpus, and a reduced 4-topic structure (K = 4) was used to summarize macro-level discourse frames.

**Results:**

Prominent themes encompassed hierarchical/relational conflict, work conditions and employment transitions (e.g., resignation, contracts, dismissal), and institutional responses (e.g., reporting, evidentiary documentation, investigation, disciplinary procedures, and legal consultation/disputes). Descriptive comparisons suggested that later-period discourse clustered more clearly around reporting, investigation, documentation, and legal advice, whereas psychosocial distress and interpersonal mistreatment remained salient across the study period.

**Conclusion:**

Post-legislation Q&A discourse on workplace bullying appears to reflect layered narratives in which lived psychosocial distress coexists with salient procedural and evidentiary concerns. These findings have implications for public health implementation, underscoring the need for integrated prevention and response systems that reduce psychosocial burden while improving the accessibility and navigability of institutional processes.

## 1 Introduction

Workplace bullying is a psychosocial hazard that cumulatively undermines workers' mental and physical health and impairs occupational functioning (1). Beyond interpersonal conflict, it constitutes a public health problem that calls for population-level prevention and intervention (2, 3). Workplace bullying is typically distinguished from isolated workplace disagreements by a pattern of repeated negative acts over time, coupled with a power imbalance that makes it difficult for the target to defend against or stop the behavior (4). Such negative acts range from overt aggression to more subtle forms of mistreatment, including exclusion, social disregard, excessive work demands, and humiliating or demeaning communication (5).

Internationally, the policy and legal landscape has increasingly recognized workplace violence and harassment—including bullying—as an institutional and regulatory concern. The International Labor Organization (ILO) Violence and Harassment Convention, 2019 (No. 190) defines violence and harassment in the world of work as a range of unacceptable behaviors and practices, or threats thereof, that are aimed at, result in, or are likely to result in physical, psychological, sexual, or economic harm—regardless of whether the conduct occurs as a single event or repeated over time (6, 7). This global framework provides a normative basis for addressing psychosocial hazards through policy and organizational systems rather than relying solely on individual coping or interpersonal resolution.

The burden of workplace bullying is evident in both prevalence and consequences. Although estimates vary by measurement approach and context, meta-analytic evidence suggests that bullying exposure is not uncommon (e.g., an average prevalence of approximately 14.6%) (8). Exposure has been linked to a range of adverse mental health outcomes, including depression, anxiety, post-traumatic stress symptoms, and sleep disturbances, as well as physical health deterioration such as cardiovascular problems, musculoskeletal symptoms, and immune dysregulation (9, 10). These health impacts can translate into prolonged sickness absence, reduced work performance, and turnover, amplifying organizational and societal costs (11, 12). At the organizational level, bullying is associated with absenteeism, turnover, productivity loss, and increased medical and management expenditures (13), and some work has estimated substantial costs at the national level (14). Taken together, these cumulative harms support the view that workplace bullying is not merely an individual adjustment issue but a public health challenge that requires multilevel prevention involving organizational, institutional, and community resources (15, 16).

Research on workplace bullying has developed along several major trajectories. First, epidemiological and meta-analytic studies have repeatedly documented associations between bullying exposure and poorer mental health and other negative outcomes (10, 12, 17). Second, organizational psychology and management research has identified antecedents such as leadership patterns, organizational justice, job stressors, and power imbalances (18, 19), thereby informing theoretical targets for prevention (20). Third, intervention studies have examined organizational policies, training programs, and reporting systems, but findings remain mixed (21), and scholars have emphasized that effectiveness may hinge on cultural and institutional context as well as implementation quality (22, 23). From a public health standpoint, therefore, the key question is not only what policies exist, but how they are enacted in practice—and how workers navigate these systems when seeking help.

As legal and policy efforts to regulate workplace bullying have expanded in many countries, interest has grown in whether regulatory changes are accompanied by shifts in reporting, organizational responses, and use of support systems. Within this broader trend, ILO Convention No. 190 and Recommendation No. 206 have promoted the institutionalization of standards for “violence- and harassment-free workplaces,” encouraging national and organizational regulatory strengthening (7, 24). Yet empirical evidence on the effects of legal reforms remains limited. Existing work has often focused on quantitative indicators of system use after legal adoption—such as complaint counts, policy awareness, or case-law analysis (25–27). A public health-relevant question extends beyond whether legal channels are used more frequently. Specifically, it asks whether and how the introduction of legal and procedural frameworks may be associated with changes in how workers label experiences as “bullying,” attribute responsibility, and seek institutional remedies—processes of case construction and proceduralization through institutional language and pathways (28, 29). Based on prior literature on workplace-bullying interventions, reporting barriers, and institutional responses to workplace mistreatment, we expected later-period discourse to place greater emphasis on reporting, investigation, and evidentiary aspects of help-seeking, while experiential suffering would remain present (30–32).

Online discourse offers a distinctive vantage point for examining such reorganization. In this study, “online discourse” refers to naturally occurring text generated when users voluntarily describe workplace problems and solicit advice on an online platform, rather than text elicited by researcher-designed survey items or interview prompts. Accordingly, the present study does not aim to estimate causal effects of legal regulation. Instead, it provides an empirical description of how workplace bullying discourse evolves and reorganizes during the implementation trajectory of a legal reform.

Discourse-oriented scholarship has contributed to understanding workplace bullying not only as interpersonal behavior but also as a phenomenon constructed within social and institutional contexts (33, 34). Qualitative research has suggested that organizational policy and procedural language can frame bullying in ways that generate conceptual ambiguity (35, 36), and that targets' narratives may depict bullying as diffuse and ongoing rather than as discrete incidents (37). Others have argued that formal procedures can impose procedural burdens and produce secondary victimization (38). However, empirical evidence on how naturally occurring help-seeking discourse reorganizes over time—particularly in relation to institutional references such as evidence, investigation processes, and medical/administrative pathways—remains scarce.

To address this gap, topic modeling is employed as a computational text analysis approach consistent with a “text-as-data” perspective (39). Importantly, the aim is not to reproduce micro-level discursive mechanisms (e.g., rhetorical moves, justification strategies, or accountability work). Rather, latent thematic structure is estimated from a large corpus, and the analysis describes how clusters of recurring issues are organized and foregrounded over time. Topic interpretation was informed by top-ranked terms and representative documents, and labels were refined iteratively to ensure conceptual clarity and internal consistency.

South Korea introduced a legal prohibition of workplace bullying and imposed duties on employers to investigate and respond through amendments to the Labor Standards Act in July 2019. Following implementation, demand for consultation and complaint filing has been reported, and public attention has expanded (40). Nevertheless, organizational contexts characterized by relatively high power distance, relational norms, and tendencies toward organizational silence may create barriers to reporting and intervention (41, 42). For example, the anonymity afforded by online platforms has provided a new outlet for employees in a hierarchical culture to share experiences and seek help without direct confrontation, amplifying voices that might otherwise be silenced (43). At the same time, Korea's relationship-oriented, face-saving norms could lead to more indirect or deferential language even in anonymous posts—victims may downplay accusations or frame their stories cautiously to avoid “losing face,” reflecting cultural pressures even in online discourse (44). Concerns have also been raised that the occupational safety and health system has historically emphasized physical hazards, potentially leaving psychosocial risk management comparatively underdeveloped, particularly amid high levels of nonstandard employment and job insecurity (45, 46). In such a context, even well-intended legal protections can have unintended effects: fear of retaliation or stigma may persist despite new formal channels, discouraging some victims from reporting. Notably, surveys indicate that a significant proportion of Korean workers who experience bullying do not officially report it, and among those who do, nearly half have faced retaliatory disadvantages (47). These patterns suggest that legal reform must be coupled with credible safety mechanisms and cultural change to truly empower workers. In this context, examining whether legal regulation is accompanied by shifts in workers' language, coping strategies, and institutional resource invocation can inform improvements in implementation—focusing on how policy is actually used and perceived, not merely its existence.

Online question-and-answer (Q&A) platforms can function as accessible, often anonymous spaces where workers narrate workplace experiences and seek advice, providing a window into unsolicited narratives that may not be captured well through official statistics or survey instruments (48). In South Korea, Naver Knowledge iN hosts a long-running archive of workplace-related questions, enabling analysis of discursive reorganization following institutional change (49). Topic modeling is an unsupervised machine-learning approach that infers latent thematic structure in large text corpora with minimal reliance on strong *a priori* assumptions (50). In public health research, topic modeling has been applied to analyze online discourse, understand health information seeking, and track policy discourse shifts (51–53).

The methodological contribution of this study lies in capturing how workers articulate experiences and invoke response resources (e.g., evidence, investigation procedures, legal consultation, medical/administrative pathways) in naturally occurring help-seeking text. Compared with surveys or interviews, which can constrain the scope of experiences by design, online narratives may more directly reflect how procedural burdens, substantiation strategies, and help-seeking pathways combine in real-world situations (48, 54). Using a large corpus accumulated between July 2019 and July 2025 also enables a descriptive examination of how procedural and event-oriented framings appear across the corpus and across periods as institutional pathways become more salient. At the same time, the analysis is conceptually and structurally bounded to experiences explicitly labeled as “workplace bullying.” This study clarifies its scope by distinguishing this term from related Korean concepts. “Gapjil” refers to an abuse of power by superiors rooted in hierarchical culture, broadly aligning with workplace bullying yet differing in cultural perception (55). “Emotional labor” refers to the imposed management of feelings in customer-facing roles, highlighting psychological stressors distinct from peer harassment (56). These data should be interpreted as an auxiliary signal that complements—rather than represents—population-level prevalence or official indicators, and any policy implications must be derived with attention to representativeness limitations and privacy/ethical principles (57). Because the corpus was assembled using “workplace bullying” as a search term, the analysis is structurally bounded to discourse in which users adopt that label; accordingly, the study aims to describe how “bullying”-named experiences are organized in relation to institutional and procedural language, rather than to estimate population-wide changes in recognition.

Workplace-bullying-related posts from Naver Knowledge iN collected between July 16, 2019, and July 31, 2025, were analyzed across two post-legislation periods: an early period (July 16, 2019—June 30, 2022) and a late period (July 1, 2022—July 31, 2025). Latent Dirichlet Allocation (LDA) topic modeling was employed to identify recurring thematic structures. Period-specific models were estimated separately and compared as descriptive thematic frames, reporting both a macro-level solution (K = 4) and a finer-grained solution (K = 12).

Accordingly, this study addresses three research questions: (1) What are the primary topics that structure online help-seeking discourse about workplace bullying, and what components constitute these topics? (2) How do topic structure and thematic salience differ between the early and late periods? (3) What implications do observed discursive patterns offer for public health prevention and intervention strategies? Drawing on the public health three-tier prevention framework—primary, secondary, and tertiary prevention—the study considers how observed discursive patterns may inform prevention and intervention priorities under policy implementation (22, 58, 59). Ultimately, the present study provides a descriptive account of how workplace-bullying discourse is organized across two post-legislation periods, offering a comparative case for settings with similar institutional and cultural conditions.

## 2 Methods

### 2.1 Research design and analytical framework

This study adopts a bottom-up, data-driven analytical framework to examine how workplace bullying is articulated and reorganized in online discourse following the introduction of legal regulation in South Korea. Rather than imposing predefined theoretical or legal categories, the analysis seeks to identify latent thematic structures emerging from large-scale, naturally occurring textual data.

**Figure 1:**
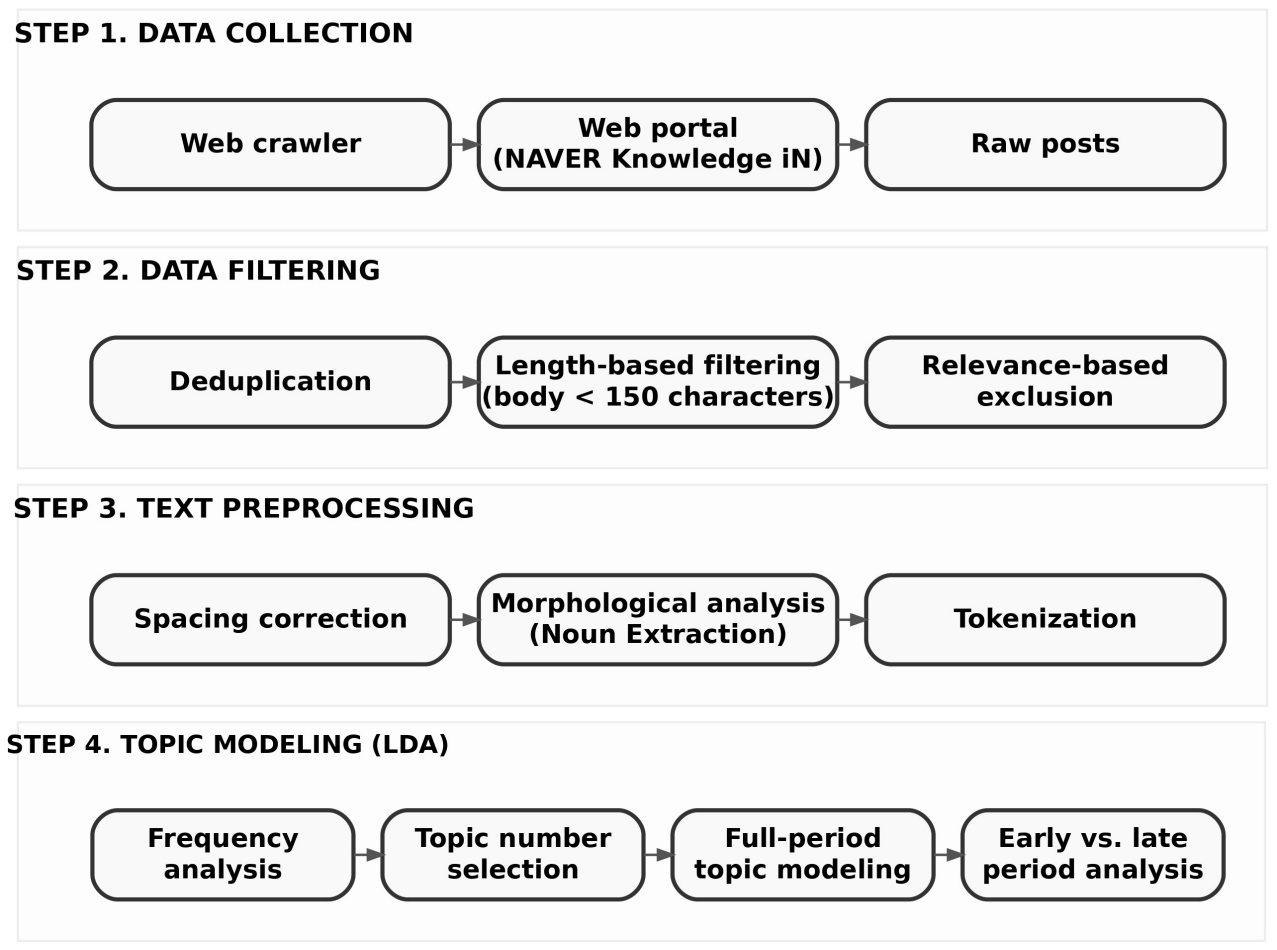
Analytical framework.

As illustrated in Figure 1, the analytical workflow consists of four sequential stages: data collection, data filtering, text preprocessing, and topic modeling. First, online posts related to workplace bullying were collected from Naver Knowledge iN using a web crawler. Second, the raw dataset was refined through deduplication, length-based filtering, and relevance screening to ensure semantic adequacy and analytical consistency. Third, the filtered texts underwent preprocessing procedures—including spacing correction, morphological analysis, and noun-based tokenization—to reduce linguistic noise while preserving the semantic characteristics of informal Korean-language discourse.

In the final stage, Latent Dirichlet Allocation (LDA) topic modeling was applied to the preprocessed corpus. Prior to topic estimation, frequency analysis was conducted to examine overall lexical distributions. In this study, frequency refers to corpus-level token frequency, namely the total number of occurrences of each token across all documents in the corpus, rather than the number of documents in which the token appears. The optimal number of topics was determined by evaluating model performance across different values of K. Topic models were then estimated for the full observation period as well as for early and late post-legislation periods, enabling both overall and temporal analyses of workplace bullying discourse.

By structuring the analysis around these interconnected stages, the research framework ensures methodological coherence and transparency, allowing systematic examination of how individual experiences are transformed into recurring discursive patterns and how these patterns differ across corpora in the context of legal regulation.

### 2.2 Data collection

Online textual data related to workplace bullying were collected from Naver Knowledge iN (https://kin.naver.com), a major question-and-answer platform in South Korea. The platform allows users to anonymously post questions and narratives regarding personal, occupational, and legal concerns, making it well suited for capturing first-person accounts and informal discourse on workplace bullying.

Posts were retrieved using the Korean-language search query for workplace bullying. The data collection period spanned from July 16, 2019 (the official enforcement date of workplace bullying regulations in South Korea) to July 31, 2025. This timeframe was selected to capture post-legislation discourse and enable temporal comparisons between the early and late post-legislation periods. Data collection was automated using a Python-based web crawler (Python version 3.13.5) in a macOS environment, implemented with Selenium, Google Chrome, and the corresponding ChromeDriver.

For each post, the title, body text, and publication date were collected. Only original question posts were included; replies and comments were excluded to maintain consistency in the unit of analysis.

Regarding ethical considerations, this study was confirmed to be exempt from IRB review by the Yonsei University Institutional Review Board (Approval Number: 7001988-202512-HR-3053-01E). The dataset consisted exclusively of publicly accessible online question posts collected from Naver Knowledge iN, and all data were obtained in compliance with the platform's terms of service. The study did not involve bypassing access restrictions, interacting with users, or collecting private user information. To reduce privacy risks, personally identifying information, including names of individuals, workplace identifiers, and contact details, was not included in the analytic dataset or reported in the manuscript. When illustrative excerpts were used, they were selected and presented cautiously so as to avoid unnecessary exposure of sensitive personal narratives and to minimize the risk of secondary victimization.

### 2.3 Data filtering

The initial crawling process yielded 23,605 posts. First, duplicate posts were identified based on identical titles, which typically indicated repeated or reposted questions on the platform. As a result, 1,610 duplicate posts were removed, leaving 21,995 unique posts.

Prior research has highlighted the limitations of relying solely on textual similarity in user-generated content and the common use of heuristic preprocessing in large-scale text analysis (39). In this study, title-based deduplication was implemented as a procedural step to remove clearly redundant entries, rather than to identify semantically equivalent content.

Next, to ensure sufficient semantic information for stable topic modeling, posts were filtered based on body text length. Prior studies have shown that very short documents provide limited word co-occurrence information, leading to unstable topic inference in probabilistic models such as LDA (60). Following prior research on online inquiry platforms and Korean text analysis (61), posts consisting only of titles (*n* = 746) and posts with body text shorter than 150 characters (*n* = 5,759) were excluded. After length-based filtering, 15,490 posts remained.

While the 150-character threshold was introduced to ensure sufficient semantic content for stable topic modeling—given that very short texts may provide limited word co-occurrence information (60, 62)—it was also informed by the nature of online help-seeking narratives. In many cases, very short posts tend to consist of fragmentary questions or keyword-based inquiries, offering limited contextual detail for interpreting workplace experiences (63). Accordingly, the length criterion was applied as a pragmatic balance between model stability and the inclusion of posts with sufficient contextual information for analysis.

Subsequently, posts were screened for relevance to workplace bullying to exclude content that fell outside the conceptual scope of the study despite containing the search keyword. To ensure conceptual clarity, explicit operational criteria were established to distinguish workplace bullying from related but distinct forms of workplace misconduct, particularly sexual harassment and workplace violence. In this study, workplace bullying was defined as repeated or sustained negative acts occurring within a workplace context that involve power imbalance and result in psychological distress, including behaviors such as verbal abuse, exclusion, humiliation, excessive monitoring, or unfair work assignments.

In contrast, sexual harassment and sexual violence were defined as behaviors involving unwanted sexual attention, sexually explicit language or actions, or coercion of a sexual nature, regardless of whether they occurred within the workplace. Although such behaviors may co-occur with workplace bullying, they are governed by distinct legal definitions and institutional response systems. Based on these definitions, posts were included if they contained substantive descriptions of non-sexual, power-related interpersonal mistreatment consistent with workplace bullying, even when other forms of misconduct were also mentioned. Conversely, posts were excluded if (a) the content focused primarily on sexual harassment or sexual violence without describing broader patterns of workplace bullying, or (b) the reference to workplace bullying was superficial or incidental (e.g., keyword mention without experiential description).

Importantly, references to sexual harassment or sexual violence were not treated as automatic exclusion criteria. Posts in which such experiences appeared as secondary or contextual elements within a broader narrative of workplace bullying were retained in the dataset. Only cases in which sexual harassment or sexual violence constituted the primary focus of the post, without substantive description of workplace bullying dynamics (e.g., power imbalance, repeated mistreatment), were excluded. This distinction allowed the analysis to capture the complex and overlapping nature of workplace experiences while maintaining conceptual focus on workplace bullying.

Based on these criteria, a multi-step filtering procedure was implemented. First, posts lacking substantive descriptions of workplace bullying were automatically excluded. Posts containing references to workplace violence or sexual harassment were identified through automated keyword screening and reviewed for relevance. Only posts lacking substantive descriptions of workplace bullying were excluded from the analytical dataset. Subsequently, the remaining posts were manually reviewed to exclude cases that did not involve the author's own experiences, such as references to third-party incidents, media reports, or broadcast content. To enhance reliability, the initial coding and filtering were conducted by one researcher and subsequently reviewed by a second researcher. Discrepancies were discussed and resolved through consensus.

**Figure 2:**
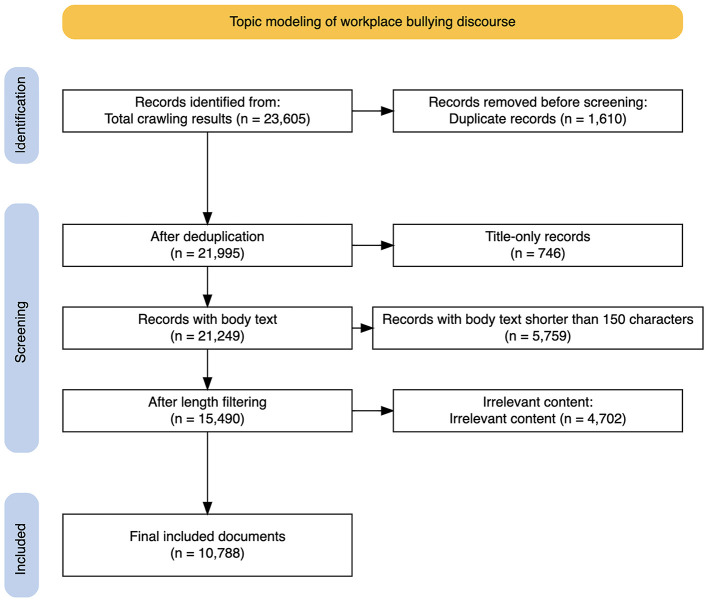
Flow diagram of data selection and filtering process.

After relevance-based filtering, an additional 4,702 posts were excluded. The final analytical dataset consisted of 10,788 posts (M = 555.48 characters, SD = 479.59). For temporal analysis, the dataset was divided into two periods: an early period from July 16, 2019 to June 30, 2022 (*n* = 4,571), and a late period from July 1, 2022 to July 31, 2025 (*n* = 6,217). A detailed flow of the data filtering and exclusion process is presented in Figure 2 (PRISMA diagram), including the number of records removed at each stage and the specific reasons for exclusion. Representative examples of included and excluded posts following the filtering process are provided in Supplementary Table S4.

### 2.4 Text preprocessing

All texts underwent a multi-stage preprocessing procedure commonly adopted in Korean natural language processing research. First, spacing correction was applied using PyKoSpacing to address inconsistencies in Korean spacing known to affect tokenization and morphological analysis (64). The corrected texts were then segmented based on whitespace.

Prior to tokenization, a corpus-level frequency list was generated and examined to support systematic preprocessing (39). Based on this list, two researchers manually examined the full set of candidate words extracted from the corpus and conducted rule-guided refinement before tokenization. In this process, expressions that could be consistently nominalized were converted into noun forms, semantically redundant or non-informative expressions were excluded, and multiple expressions referring to overlapping meanings—particularly those conceptually related to workplace bullying—were unified into standardized lexical forms. Through this manual review, recurrent patterns in meaningless, redundant, or semantically overlapping expressions were identified and formalized into preprocessing rules.

These rules were organized into a domain-specific lexicon and applied prior to tokenization. Based on this lexicon, stopwords were defined and normalization rules were implemented to consolidate spelling variants and semantically equivalent expressions, thereby reducing lexical redundancy and improving semantic consistency (39). In addition, rule-based post-processing was applied to merge frequent compound nouns into single tokens (e.g., “unemployment benefits,” “workplace bullying”), minimizing lexical fragmentation. Detailed lists of the domain-specific lexicon, stopword entries, normalization rules, and token-combination procedures are provided in Supplementary Tables S1–S3, together with representative examples of lexical standardization. This documentation is provided to enhance transparency in preprocessing decisions and to reduce the possibility that the identified topic structure reflects arbitrary researcher-driven choices. To further assess the robustness of preprocessing decisions, sensitivity analyses were conducted using alternative preprocessing specifications, including retaining stopwords and omitting normalization rules. All models were estimated under identical modeling parameters. As shown in Supplementary Table S5, coherence scores ranged from 0.439 to 0.472 across conditions, while log perplexity remained stable. Although the removal of stopwords improved topic coherence, the overall topic structure and interpretability were largely consistent across preprocessing conditions, indicating that the identified thematic patterns were not driven by specific preprocessing choices (65). These findings suggest that the identified topic structure was robust to reasonable variations in preprocessing decisions.

For descriptive word-frequency analysis, query-defining keywords and their close variants (e.g., “workplace bullying,” “bullying”), as well as platform-redundant generic terms (e.g., “company,” “workplace”), were excluded from frequency counts to avoid trivial repetition. Importantly, the same lexicon-based preprocessing pipeline and resulting corpus were used consistently for both word-frequency analysis and subsequent topic modeling.

Morphological analysis and tokenization were conducted using the KOMORAN analyzer via KoNLPy. Tokenization was restricted to nouns (NN^*^ tags), which are considered most informative for representing document-level semantic content in Korean text, while reducing syntactic and functional noise commonly introduced by particles and verbal endings (66, 67). Prior studies have shown that noun-focused representations improve topic coherence and interpretability by concentrating on core semantic units of documents (67, 68).

However, restricting tokens to nouns may lead to the loss of evaluative or affective information, which is typically conveyed through adjectives, adverbs, and verbal expressions (69). To mitigate this limitation, the preprocessing pipeline incorporated a rule-based normalization procedure in which evaluative expressions were systematically nominalized or mapped to semantically equivalent noun forms wherever possible. In addition, domain-specific lexicon construction and manual review ensured that frequently occurring evaluative meanings—particularly those relevant to workplace bullying experiences, where such evaluations play a central role (38)—were preserved in standardized lexical representations.

Furthermore, topic interpretation was not based solely on token distributions but also involved iterative examination of representative documents for each topic. This procedure allowed evaluative and contextual meanings, which may not be fully captured by noun-only tokens, to be reintegrated during the interpretative phase. These strategies enabled the advantages of noun-based representations to be retained while mitigating potential loss of evaluative and contextual information during analysis (67, 68).

### 2.5 Latent Dirichlet allocation topic modeling

Latent Dirichlet Allocation (LDA) was employed to identify latent thematic structures in workplace bullying discourse. LDA models documents as mixtures of topics and topics as distributions over words, allowing thematic interpretation based on word co-occurrence patterns (50).

**Figure 3:**
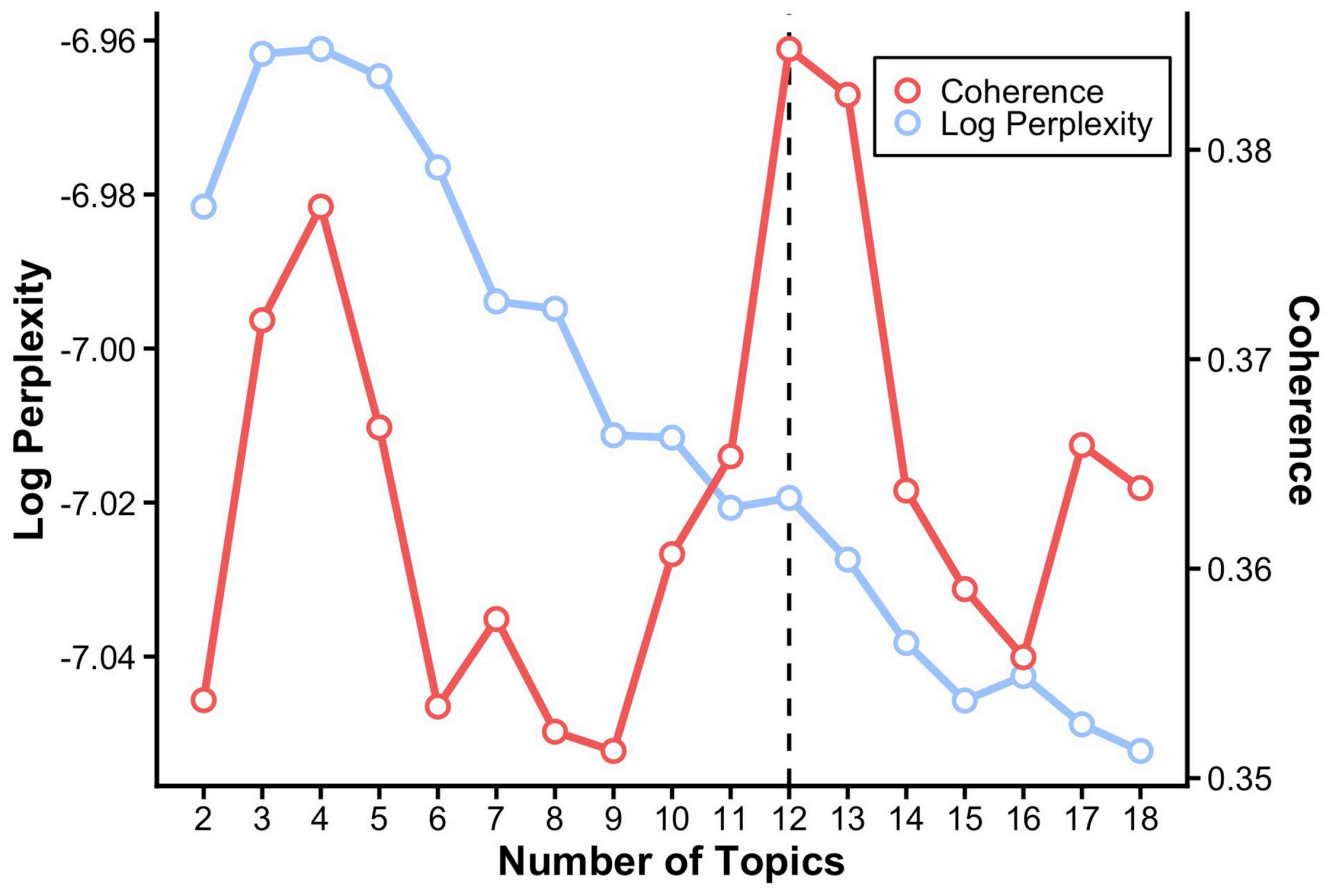
Topic coherence and perplexity across different numbers of topics.

Topic modeling was conducted using a Bag-of-Words document–term matrix. To determine the optimal number of topics, LDA models were estimated with topic numbers (K) ranging from 2 to 18 under identical preprocessing and learning conditions. Model performance was evaluated using perplexity and topic coherence (c_v and c_npmi). Consistent with prior topic modeling research, topic coherence was treated as the primary criterion for selecting the number of topics, as it reflects the semantic interpretability and internal consistency of topic-word distributions, whereas perplexity was used as a supplementary diagnostic indicator of model fit. The full range of coherence and perplexity values across K = 2–18 is visualized in Figure 3, allowing direct comparison of model performance across different topic numbers.

Perplexity was calculated in log scale on a held-out test set obtained via an 80:20 train-test split of the corpus, with the split fixed using a random seed (seed = 42). All LDA models were trained using identical hyperparameters (passes = 10, iterations = 200, chunksize = 2,000, random_state = 42) to ensure comparability across different values of K. Based on the joint examination of coherence (c_v and c_npmi) and log perplexity metrics, and prioritizing semantic interpretability, a 12-topic solution was selected for subsequent analyses. Specifically, c_v coherence reached its highest value at K = 12 (0.3848) and decreased slightly at K = 13 (0.3826). Although c_npmi coherence was marginally higher at K = 13 than at K = 12 (0.0116 vs. 0.0111), this difference was minimal. Log perplexity was slightly lower at K = 13 than at K = 12 (−7.0274 vs. −7.0194), indicating a marginal improvement in statistical fit; however, this gain was limited and accompanied by reduced semantic coherence. Importantly, visual inspection of coherence and perplexity trends indicated a stabilization point around K = 12, beyond which improvements in model fit were marginal while interpretability decreased. Taken together, these results indicate that K = 12 provided the best balance between semantic coherence, interpretability, and model parsimony. These results are reported in Supplementary Table S6.

To examine temporal variation, the same modeling procedure was applied separately to the early and late periods. For each period, topic models with K = 4 were estimated to capture macro-level discourse patterns, alongside models with K = 12 to explore finer-grained thematic structures. Topic interpretation was based on the combined examination of top-ranked words and representative documents, with topic labels assigned through iterative researcher discussion to ensure conceptual clarity and internal consistency.

To examine temporal variation in topic prevalence, yearly topic proportions were calculated by averaging document-level topic distributions within each year. These aggregated values were used to visualize temporal trends and identify topics with the largest changes over time. The resulting yearly topic proportions were used to generate visualizations presented in the Results section.

## 3 Results

### 3.1 Word frequency analysis

Core search terms such as workplace bullying and closely related expressions appeared with high frequency across the corpus; however, these terms were excluded during preprocessing and therefore did not appear in the reported frequency analysis to avoid trivial repetition. After exclusion, the most frequently occurring words primarily reflected workplace roles and organizational contexts (e.g., work, employee, supervisor, company), as well as experiential and psychological states (e.g., stress, distress, mental health).

In addition, relational and employment-related terms—including resignation, report, dismissal, and unemployment benefits—ranked highly, indicating that workplace bullying discourse was closely intertwined with both interpersonal dynamics and broader employment-related concerns. Notably, terms associated with institutional processes (e.g., evidence, legal action, human resources, investigation) also appeared frequently. Table 1 presents the frequencies of these top-ranked words, illustrating the prominence of terms related to organizational contexts and reporting processes.

**Table 1:** Frequencies of the top ranked words.

Rank	Word	Frequency	Rank	Word	Frequency
1	Work	9,352	21	Verbal abuse	2,084
2	Resignation	9,040	22	Perpetrator	2,050
3	Employee	9,018	23	Leaving work	2,016
4	Report	8,712	24	Writing	1,950
5	Working conditions	6,982	25	Phone call	1,936
6	Supervisor	5,472	26	Unfairness	1,928
7	Unemployment benefits	3,540	27	Coworker	1,881
8	Distress	3,471	28	Handling	1,876
9	Team leader	3,246	29	Method	1,837
10	Evidence	3,046	30	Instruction	1,780
11	Going to work	2,690	31	Reason	1,776
12	Employer	2,679	32	Dismissal	1,754
13	Stress	2,598	33	Recognition	1,751
14	Human resources	2,554	34	Individual	1,727
15	Legal action	2,547	35	Insults	1,722
16	Department	2,435	36	Seat	1,690
17	CEO	2,325	37	Application	1,664
18	Mental health	2,234	38	Process	1,661
19	Hiring	2,228	39	Contract	1,660
20	Audio recording	2,148	40	Afterward	1,653

### 3.2 Determination of the number of topics

Figure 3 illustrates the changes in topic coherence and perplexity across different numbers of topics, ranging from 2 to 18. Topic coherence was used as the primary criterion for selecting the optimal number of topics, as higher coherence values indicate greater semantic interpretability and internal consistency of the extracted topics. Perplexity values are reported in log scale and are presented as a comparative indicator of model fit rather than as an absolute criterion for topic selection.

As shown in Figure 3, coherence scores did not increase monotonically with the number of topics but instead exhibited noticeable fluctuations across different model specifications. Notably, a clear local maximum in coherence was observed at K = 12. Beyond this point, coherence scores either declined or varied without demonstrating a consistent or substantial improvement. While models with fewer topics resulted in overly broad and less differentiated thematic structures, models with a larger number of topics tended to fragment semantically related themes, reducing interpretability.

Log perplexity values decreased as the number of topics increased, indicating improved model fit with greater model complexity. However, consistent with prior topic modeling studies, perplexity was treated as a supplementary diagnostic measure, given its tendency to favor larger topic numbers without necessarily improving semantic clarity. Taken together, these results suggest that a 12-topic solution provides an appropriate balance between model fit and semantic interpretability. Accordingly, subsequent analyses were conducted using an LDA model with 12 topics.

### 3.3 Overall topic structure of workplace bullying discourse

**Figure 4:**
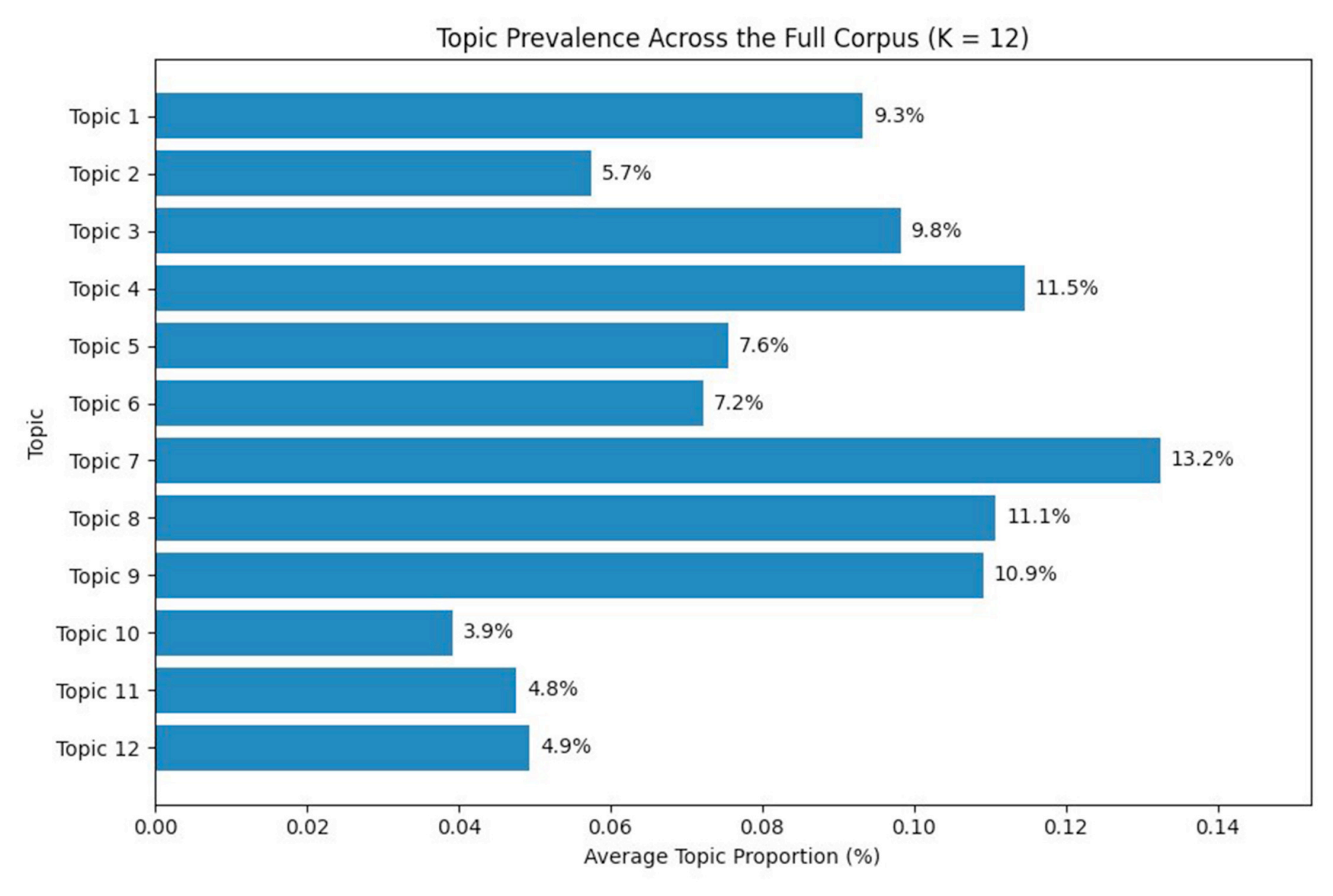
Topic prevalence across the full corpus (K = 12).

Using the selected model with K = 12, topic modeling was applied to the full dataset to identify the overall thematic structure of workplace bullying discourse (see Table 2). The resulting topics captured a broad range of experiences and concerns, encompassing interpersonal conflict, organizational hierarchy, employment instability, legal procedures, and psychological distress. The relative prevalence of each topic across the corpus is presented in Figure 4.

**Table 2:** Representative topics and illustrative excerpts from online workplace bullying discourse.

Topic	Topic label	Representative keywords	Representative excerpts for each topic
1	Attendance, leave, and resignation conflicts in small businesses and part-time work	leave, employer, part-time work, resignation letter, attendance, vacation, absence, store, manager	“After submitting my resignation, I requested to use my remaining annual leave, but the company rejected it, citing the absence of a replacement. Would this situation be considered workplace bullying?”
2	Organizational hierarchy and resignation-related conflicts in office settings	employee, CEO, resignation, executive, task handover, office, hiring, company dinner	“A senior employee repeatedly assigns me non-work-related personal errands, justifying it by citing the difference in rank. When I expressed discomfort, I was told that such tasks were part of my role and ordered to comply. Would this type of directive be considered workplace bullying?”
3	Legal disputes involving harassment, threats, and compensation claims	lawsuit, civil case, sexual harassment, claim, legal complaint, lawyer, compensation, threat	“After reporting workplace bullying to the labor authorities, I am considering filing a civil or criminal lawsuit. Would a confirmed ruling increase my chances of success, and what are the typical costs of hiring a lawyer?”
4	Dismissal and labor condition disputes (wages, contracts, severance)	dismissal, contract, wages, unfair treatment, severance pay, employment contract	“I worked without a written contract and was not clearly informed of my working conditions. Although a 1-hour break was stated, I often received little or no actual rest. After submitting my resignation, I experienced what I perceived as intentional mistreatment. Would this situation be subject to reporting?”
5	Reporting verbal abuse and evidence collection in bullying incidents	report, verbal abuse, evidence, supervisor, recording, insult, assault	“During a work-related gathering, my supervisor suddenly hit me on the back during a work-related gathering, which is clearly captured in an audio recording. When I reacted by pushing back, the situation escalated. Given these circumstances, would it be possible to file charges for assault?”
6	Excessive workload and coercive work demands	work assignment, overtime, weekend work, coercion, workload, supervision	“Even after completing my assigned work, I am pressured to stay longer due to implicit expectations from other teams. I am also expected to come in on weekends when other teams are busy. Would these situations be considered workplace bullying?”
7	Managerial surveillance and organizational control mechanisms	manager, surveillance, CCTV, mobile phone, monitoring, control, union	“To report workplace bullying, I am considering recording conversations that I am directly involved in. Would such recordings be legally permissible, and could they be shared with a third party for reporting purposes? If not, would obtaining consent from the other party make it permissible to disclose the recordings?”
8	Formal investigation and administrative procedures following reports	report, investigation, perpetrator, victim, labor office, disciplinary action	“After reporting workplace bullying, the case was dismissed without disciplinary action. I find this difficult to accept. What options are available to appeal this decision?”
9	Team-level management and task allocation conflicts	team leader, department, personnel transfer, task allocation, meetings, reporting	“Since last year, my supervisor has been excluding me from important meetings and withholding work-related information, making it difficult for me to perform effectively. I have also received the lowest performance evaluation and have been socially isolated from colleagues. Would this situation be considered workplace bullying?”
10	Employment stability, contract conditions, and leave-related issues	contract period, probation, salary, maternity leave, return to work, employment status	“About a month before the end of my contract, my supervisor began pressuring me to resign, stating that either I should leave or they would. The situation has made it difficult to continue working, and several employees have already left due to similar treatment. What options do I have to respond to this situation?”
11	Emotional distress during early employment and workplace adaptation	stress, distress, mistake, being ignored, anxiety, emotional difficulty	“I am experiencing workplace bullying, including the spread of negative rumors about me. Although I try to ignore the situation to avoid conflict, the stress has become overwhelming, and I find myself experiencing severe emotional distress, including frequent episodes of crying. As I feel unable to leave my job or confront the issue directly, how can I cope with this depression?”
12	Post-resignation administrative and mental health support processes	resignation, unemployment benefits, industrial accident, treatment, depression	“After resigning due to workplace bullying that was later recognized by the labor authorities, I briefly worked at another company and am now considering applying for unemployment benefits. However, I understand that eligibility is based on the most recent employment. In this case, would I still qualify for unemployment benefits?”

**Figure 5:**
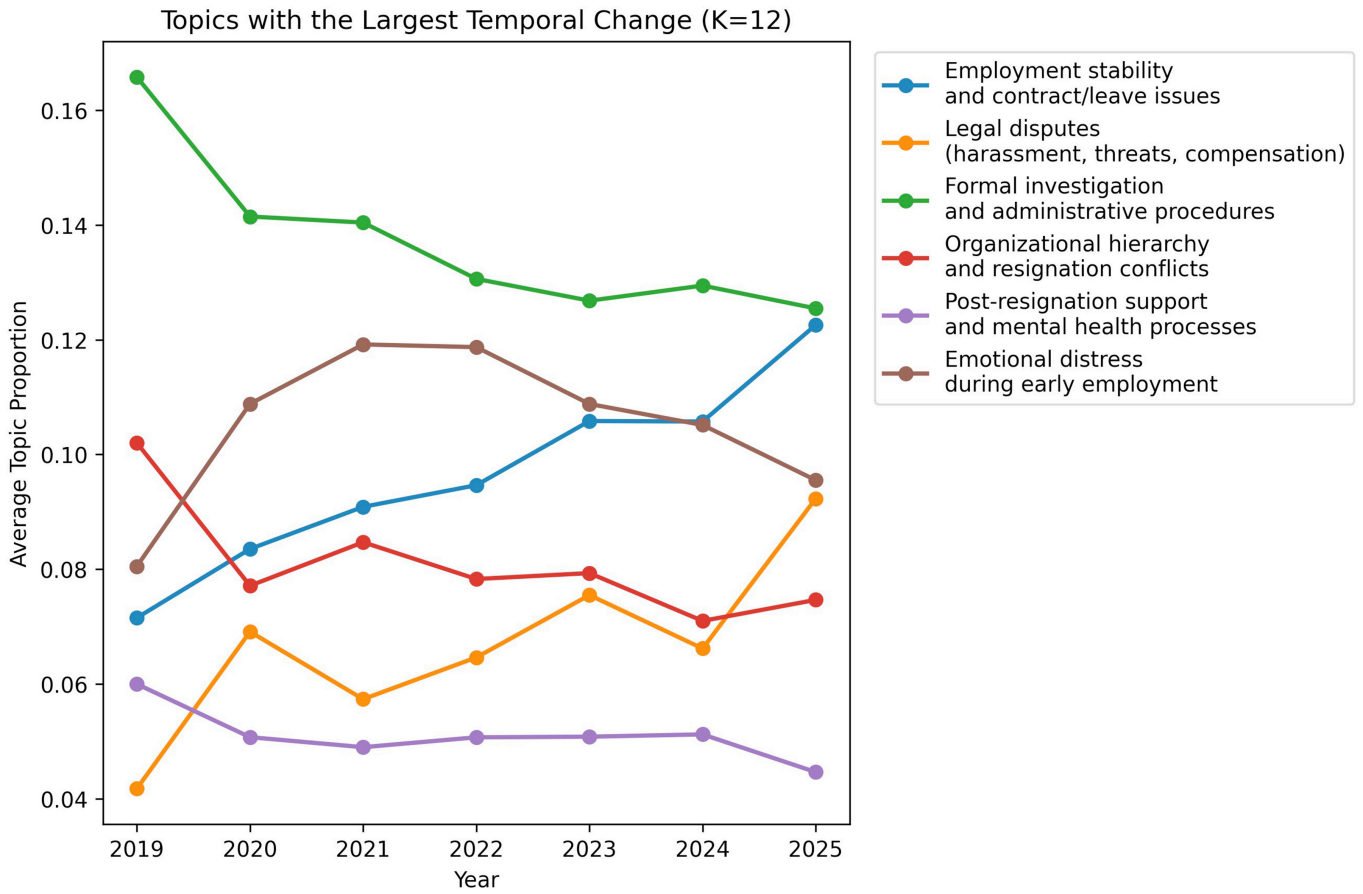
Temporal trends in selected workplace bullying topics (K = 12). The figure shows the six topics with the largest absolute changes in yearly prevalence. Values represent average document-level topic proportions.

**Figure 6:**
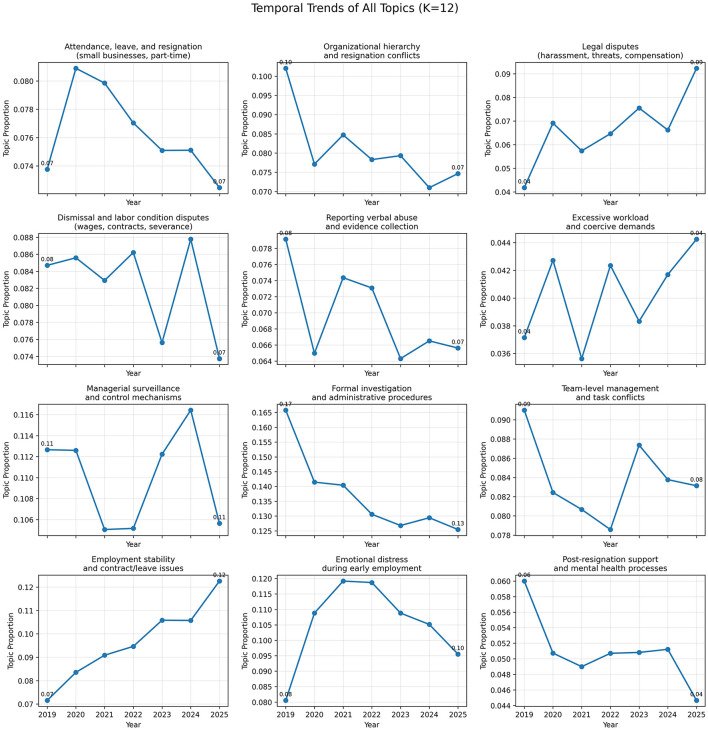
Yearly topic prevalence across all topics (K = 12). Each panel shows the yearly prevalence of a topic, calculated as the average document-level topic proportion.

To further examine temporal variation in topic prevalence, Figure 5 presents the six topics with the largest absolute changes across years. In addition, Figure 6 displays yearly prevalence trends for all 12 topics, allowing a comprehensive comparison of temporal patterns across the full topic structure. The full yearly topic prevalence values are provided in Supplementary Table S7. These visualizations suggest temporal differentiation across topics, with some topics showing steady increases, others declining after initial peaks, and several remaining relatively stable over time.

Across topics, several recurring thematic dimensions were observed. First, multiple topics centered on hierarchical power relations, including conflicts with supervisors, team leaders, and employers. Second, institutional and procedural responses—such as reporting, evidence collection, legal action, and human resources processes—formed a distinct thematic cluster, indicating that institutional and procedural language was salient in the corpus. Third, employment-related transitions, including resignation, dismissal, unemployment benefits, and contract issues, emerged as salient themes, indicating that bullying was frequently discussed in relation to career disruption. Finally, psychological and emotional experiences, such as stress, mental exhaustion, and distress, were embedded across multiple topics rather than isolated in a single theme.

To complement the yearly topic-prevalence plots, temporal trend analysis further suggested that certain topics displayed differing prevalence patterns across years. For example, topics related to employment stability and legal disputes appeared more prevalent in later years of the corpus, whereas topics related to formal investigation procedures appeared to decline after the initial post-legislation period. In contrast, some topics, such as emotional distress and managerial control, remained relatively stable, indicating persistent experiential concerns that were not substantially altered by institutional changes. These patterns may reflect both gradual institutional adaptation and enduring experiential dimensions in workplace bullying discourse over time.

### 3.4 Temporal comparison of topic distributions

To explore how workplace bullying discourse evolved following the implementation of legal regulation, topic modeling was conducted separately for the early period (July 16, 2019–June 30, 2022) and the late period (July 1, 2022–July 31, 2025). Because topic models were estimated independently for each period, the temporal comparison presented here is intended as an exploratory and descriptive contrast of thematic patterns, rather than a formal statistical test of quantitative changes in identical topics across periods. A reduced topic structure (K = 4) was used to summarize macro-level shifts in dominant discourse frames, while a more detailed structure (K = 12) was employed to examine finer-grained thematic configurations.

At the macro level (K = 4), descriptive differences in dominant discourse frames were identified between the two corpora (see Table 3). In the early period, discourse appeared to be more strongly oriented toward relational and experiential dimensions, including emotional suffering in hierarchical relationships and conflicts embedded in everyday labor conditions. Although institutional elements such as reporting and legal claims were present, they were often intertwined with resignation and post-employment uncertainty rather than articulated as standardized procedures. In contrast, in the late period, macro-level topics appeared more clearly differentiated around institutional and legal processes. Reporting, investigation, disciplinary actions, and juridical disputes appeared more visibly differentiated in the later-period corpus, suggesting that bullying experiences were more often articulated through procedural and system-oriented lenses. At the same time, a topic centered on everyday relational stress remained visible, indicating that experiential suffering continued to coexist with institutionalized framings rather than being fully replaced by them.

**Table 3:** Macro-level topic frames in the early (2019–2022) and late (2022–2025) periods (K = 4).

Topic	Early period (July 16, 2019–June 30, 2022)	Late period (July 1, 2022–July 31, 2025)
Topic 0	Post-incident responses linked to resignation, in which reporting, evidence collection, and legal claims are closely tied to unemployment benefits and job exit	Formal investigation and disciplinary procedures, framing bullying through standardized institutional processes such as investigation, sanctions, and administrative or legal resolution
Topic 1	Organizational hierarchy and managerial control, with conflicts embedded in formal structures such as departments, supervisors, and HR authority rather than emotional suffering or legal action	Employment termination and post-resignation issues, where reporting is strongly entangled with resignation, unemployment benefits, and administrative concerns following job exit
Topic 2	Relational and emotional experiences of bullying, characterized by accumulated emotional distress in hierarchical relationships prior to formal institutionalization	Legal dispute and evidentiary framing, articulating bullying as a legally contestable case requiring evidence, recordings, and juridical interpretation
Topic 3	Labor conditions and contractual conflicts, linking bullying to everyday employment issues such as contracts, wages, working hours, and leave	Persistent everyday relational stress, indicating that daily relational strain and emotional suffering remain salient despite institutionalization

Topics are contrasted as descriptive thematic frames rather than one-to-one matched categories across identical models.

At the micro level (K = 12), these macro-level patterns were further elaborated through more specific thematic distinctions (see Table 4). Topics related to legal action, organizational procedures, and employment termination appeared more salient in the late period, whereas themes centered on emotional distress and relational conflict were relatively more pronounced in the early period. Together, the K = 4 and K = 12 results suggest that workplace-bullying discourse in the later-period corpus was organized in a more layered way, while experiential narratives remained present.

**Table 4:** Micro-level topic frames in the early (2019–2022) and late (2022–2025) periods (K = 12).

Topic	Early period (July 16, 2019–June 30, 2022)	Late period (July 1, 2022–July 31, 2025)
Topic 0	Work continuation and exit process surrounding resignation, including attendance, overtime, handover, and pressure to remain before leaving	Formal investigation and disciplinary procedures following reports, involving victims, perpetrators, HR, labor authorities, and litigation.
Topic 1	Organizational operation issues related to department transfer, personnel assignment, leave, and workplace placement	Medical and psychological consequences of bullying, including treatment, diagnosis, and industrial accident compensation
Topic 2	Relational strain and emotional endurance in education and care-related occupations (e.g., teachers, instructors, caregivers)	Criminal and legal disputes such as defamation, insults, threats, and police involvement following reports
Topic 3	Employment contracts and job security issues involving regular, probationary, or fixed-term positions	Everyday managerial pressure and control by supervisors, particularly toward newcomers or subordinates
Topic 4	Conflicts arising from top-level management directives and reporting within organizational hierarchies	Labor condition disputes related to contracts, wages, leave, and reporting mechanisms
Topic 5	Daily power struggles centered on supervisors' task assignments and authority in routine work	Dismissal and wage settlement issues following termination, including unpaid salaries, and contract disputes
Topic 6	Working conditions in hospitals and field-based occupations, including shifts, night work, and on-site management	Organizational operation conflicts related to task assignment, department structure, and managerial authority
Topic 7	Early-stage everyday bullying experiences marked by emotional reactions and minor incidents	Administrative processing of resignation, including unemployment benefits and voluntary or forced departure
Topic 8	Interpersonal bullying and emotional distress involving ignoring, gossip, and relational exclusion	Relational conflict and emotional exhaustion involving coworkers, supervisors, and part-time workers
Topic 9	Formal legal responses to bullying, including reporting, lawsuits, punishment, and labor office involvement	Sexual harassment and assault cases, accompanied by legal consultation and counseling support
Topic 10	Wage, leave, and dismissal disputes between owners and, workers, particularly in small workplaces	Managerial surveillance and control over attendance, communication, and on-site work processes
Topic 11	Post-resignation institutional responses, including unemployment benefits and compensation systems	Evidence collection and documentation practices, such as recordings, emails, reports, and privacy-related issues

Topics are contrasted as descriptive thematic frames rather than one-to-one matched categories across identical models.

Overall, the K = 4 and K = 12 analyses capture complementary levels of corpus-level thematic contrast. The macro-level results (K = 4) are consistent with a contrast between more loosely organized accounts of work stress and relational conflict in the early period to more procedurally structured, report-centered framing in the late period. The micro-level results (K = 12) further indicate that, particularly in the later-period corpus, bullying-related discussions were represented across more specific issue domains such as investigation, legal disputes, and evidence documentation, while experiential distress remained present.

## 4 Discussion

Using a corpus of *N* = 10,788 workplace-bullying-related posts from Naver Knowledge iN (July 2019—July 2025), this study provides an exploratory description of how online help-seeking discourse is organized into recurring thematic structures following legal regulation. Because topic models were fitted separately by period, the analyses do not test statistical changes in identical topics or estimate causal effects. Within these constraints, the findings indicate that workplace bullying discourse extends beyond relational mistreatment and is closely intertwined with institutional references, including reporting and investigation procedures, disciplinary actions, legal disputes, evidentiary documentation, employment transitions, and medical and mental health responses.

Across periods, later discourse showed stronger clustering of procedural and institutional vocabulary—such as reporting, investigation, discipline, legal disputes, and documentation—together with clearer differentiation of thematic frames. In the 4-topic solution, period-specific discourse could be summarized along higher-order axes reflecting (a) hierarchical power relations, (b) institutional and procedural responses, (c) employment-related transitions, (d) psychological and emotional experiences. In the 12-topic solution, particularly in the later period, more granular issues—investigation processes, legal disputes, evidentiary framing and documentation, and medical or administrative responses—emerged as distinct topic domains rather than remaining embedded within broader narratives. Psychological distress was organized alongside relational and everyday stress narratives at the higher-order level (K = 4), whereas the finer-grained model (K = 12) additionally revealed more differentiated emotion- and relationship-conflict topics, suggesting a layered structure in which “suffering” and “procedural response” co-occur within the broader discourse. Notably, alongside emotional expressions (e.g., distress and stress), evaluative and action-oriented terms related to judgment, substantiation, and case handling were more visible in the later-period corpus, suggesting a stronger procedural framing of help-seeking narratives. This pattern should be interpreted as exploratory and descriptive rather than inferential.

### 4.1 Interpretation in relation to prior literature

International evidence has consistently linked workplace bullying exposure to poorer mental health and adverse occupational outcomes [e.g., (10, 12)], and longitudinal studies have further connected bullying with sickness absence and related indicators [e.g., (70)]. Research on antecedents has emphasized unfavorable psychosocial work environments and destructive or laissez-faire leadership as key predictors [e.g., (13, 71)]. However, much of this literature conceptualizes bullying primarily as an exposure or outcome, paying comparatively less attention to how institutional environments—legal rules, procedures, and formal channels—intersect with the construction and narration of bullying experiences. Acknowledging that some studies do consider psychological or contextual factors indirectly, this study's unique contribution is to examine how workplace-bullying experiences are constructed and narrated within a legal regulatory context. In other words, rather than simply identifying individual-level risk factors or outcomes, the analysis adopts a discourse- and institution-sensitive perspective, exploring the social and linguistic organization of bullying narratives under a new legal regime. This approach complements the existing “antecedents–consequences” framework by suggesting that definitions of bullying, narrative forms of experience, and invoked response resources may be articulated in relation to procedural concepts such as investigation, documentation, and evidentiary substantiation. Rather than indicating direct policy effects, these patterns are more appropriately interpreted as corpus-level contrasts that bring greater attention to macro-level legal and policy contexts alongside micro-level variables. This finding is consistent with theorizing that organizational contexts characterized by silence and power distance can constrain voice and increase the burdens associated with entering formal channels, including the perceived need to generate records and proof (72).

### 4.2 Discursive reorganization: eventification and proceduralization

One descriptive contrast between the two corpora was that, in the later-period corpus, terms and frames associated with reporting, investigation, discipline, legal disputes, and evidentiary documentation appeared more salient and more clearly differentiated. This pattern should be interpreted as a descriptive contrast in how thematic frames are organized across corpora, rather than as a reconstruction of individual case trajectories. Because LDA identifies corpus-level co-occurrence patterns rather than within-case sequences, the present findings do not show how individual experiences changed over time. Instead, they suggest that the later-period corpus was more often articulated through procedural and institutional categories, with clearer visibility of documentation, investigation, and formal handling frames. These contrasts may reflect a combination of legal, organizational, and discursive conditions, but they should not be interpreted as direct evidence of causal policy effects.

The appearance of “sexual harassment and assault cases” as a distinct later-period topic, together with legal consultation and support-seeking language, further indicates that discourse labeled as “workplace bullying” may internally differentiate into subdomains with distinct institutional pathways. Importantly, references to sexual harassment or sexual violence were initially identified through automated keyword screening during data filtering and subsequently subjected to manual review. Posts were excluded only when such references appeared without substantive descriptions of workplace bullying experiences. This topic should therefore be interpreted as reflecting boundary references within “workplace bullying”-named narratives rather than as a comprehensive representation of sexual harassment discourse. These changes should not be attributed solely to legal reform; they likely also reflect broader social learning, media attention, and the diffusion of organizational rules and training. Nonetheless, the observed thematic differentiation underscores the practical importance of implementation: institutional systems shape not only whether individuals report but also how they formulate and frame their experiences when seeking help.

### 4.3 Public health implications: multilevel prevention and implementation quality

A public health approach to workplace bullying emphasizes coordinated prevention and response across levels—reducing exposure (primary prevention), enabling early detection and protective intervention (secondary prevention), and supporting treatment, recovery, and return-to-work (tertiary prevention) (58). The higher-order thematic axes identified in the 4-topic solution (hierarchical power relations–institutional and procedural responses–employment-related transitions–psychological and emotional experiences) provide a useful lens for mapping discursive concerns onto this prevention continuum and for identifying points at which burdens and resource invocation become most salient. These implications should be understood as practical interpretations of corpus-level discourse patterns rather than as direct evidence that legal regulation itself produced the observed thematic contrasts.

#### 4.3.1 Primary prevention: organizational design to reduce bullying occurrence

Recurring depictions of hierarchical control and coercive work assignment reinforce the view that bullying can be embedded in organizational structures and management practices rather than reflecting isolated individual deviance (13, 73). Primary prevention therefore targets structural risks through work design (e.g., workload, autonomy, role clarity), leadership development (communication and conflict management), and organizational culture monitoring to detect early signs of power abuse and exclusion. Given that psychosocial risk exposures may be elevated among precariously employed workers and in small workplaces or service and care sectors, prevention resources should be strategically prioritized to promote health equity (74, 75). In the South Korea context, translating these strategies into practices in small and medium-sized enterprises (SMEs) and fragmented subcontracting structures may require low-burden, scalable tools (e.g., standardized risk checklists, role-clarity templates, and brief supervisor coaching modules) rather than resource-intensive bespoke programs. Policy levers such as government subsidized consulting vouchers for SMEs and sector-specific minimum standards (e.g., for healthcare, hospitality, and care work) could operationalize primary prevention while reducing implementation gaps across organization size and employment type.

#### 4.3.2 Secondary prevention: safe reporting and high-quality investigation

The prominence of reporting, investigation, evidence, and recording within the topic structure reflects both the availability of formal channels and the potential burdens these channels impose on targets. The emergence of evidentiary documentation as an independent theme suggests that substantiation work may itself constitute a substantial demand placed on help-seekers (37, 76). Effective secondary prevention therefore requires not only formal procedures but credible safety and protection mechanisms, including physical and work-related separation during investigations, explicit anti-retaliation measures, and investigator neutrality and competence. This is particularly salient in South Korea, where survey-based evidence indicates that many targets do not report, and retaliation following reporting remains a nontrivial concern (27, 76). Providing multiple entry points—such as anonymous consultation, informal resolution options, or ombuds systems—may increase opportunities to address problems before escalation (77). To strengthen neutrality and trust, organizations can adopt independent investigation arrangements (e.g., external investigators or cross-organizational panels), mandate training/certification for internal investigators, and implement auditable anti-retaliation protocols (e.g., rapid protection orders, documentation of transfer decisions, and follow-up checks). Importantly, implementation-focused indicators are needed to assess whether procedures operate safely and effectively (e.g., time to investigation initiation, protection enactment, retaliation monitoring, investigator capacity, and recurrence following case closure), consistent with process evaluation and implementation outcome frameworks (78, 79).

#### 4.3.3 Tertiary prevention: treatment, compensation, and employment recovery support

The emergence of medical and mental health terms as a distinct later-period topic underscores that bullying-related harm may reach levels requiring clinical intervention (10, 12). Tertiary prevention includes timely linkage to mental health services (e.g., EAP, compensation pathways, treatment support), employment protection (e.g., preventing unfair dismissal and respecting worker preferences in transfers), appropriate perpetrator sanctions and recurrence prevention, and ongoing monitoring during return-to-work. Given that employment transition (resignation, dismissal, unemployment benefits, disputes) appeared as a higher-order axis, recovery support should integrate clinical care with employment, and administrative pathways rather than treating them as separate domains. Recent Korean administrative data also suggest that bullying-related harms are increasingly entering formal compensation and support systems, including work-related mental disorders recognized as industrial accidents (80, 81) and rising utilization of occupational trauma counseling services (82). These patterns underscore a practical gap that may be better addressed by case-management-style linkage integrating clinical care, documentation support, and employment/benefits navigation into a single, coordinated pathway.

#### 4.3.4 Online platforms as auxiliary public health signals

Online Q&A spaces such as Naver Knowledge iN may function as complementary help-seeking routes rather than substitutes for formal administrative, legal, or medical channels, leveraging accessibility and anonymity (48, 83). Systematic analysis of these digital traces may help identify emerging burdens (e.g., documentation pressure), procedural bottlenecks (e.g., investigation and protection measures), and distress signals among potentially vulnerable groups, thereby supplementing public health surveillance. At the same time, the use of online discourse as an auxiliary signal requires careful attention to representativeness, privacy, and ethical constraints. Importantly, these signals are shaped by selection and participation processes specific to digital platforms. The corpus is more likely to reflect workers who are digitally connected, willing to disclose experiences in semi-public anonymous settings, and motivated to seek procedural or informational guidance, while underrepresenting workers whose experiences remain offline, unlabeled, or less easily articulated in platform-based formats.

A feasible integration framework is to treat platform signals as “rapid, ground-up feedback” consistent with infodemiology/infoveillance approaches—useful for early detection of new problem forms and procedural pain points—followed by validation and targeted follow-up using conventional surveys, administrative records, or workplace audits (54, 57). In practice, spikes in specific procedural concerns (e.g., documentation pressure or delayed investigations) could trigger focused implementation reviews (e.g., response-time audits, protection enactment checks) rather than being interpreted as prevalence estimates, preserving the auxiliary-signal logic while enhancing policy responsiveness.

Taken together, these implications may extend beyond the Korean context. As jurisdictions introduce or strengthen workplace bullying regulations, help-seeking discourse may in some contexts show greater visibility of substantiation, investigation processes, and documentation, suggesting that policy effectiveness depends not only on compliance but also on implementation designs that prioritize safety, protection, and recovery (23). At the same time, discursive patterns and intervention feasibility are likely to vary across cultural and institutional contexts (22, 42). Employment arrangements (standard vs. nonstandard work), sectoral conditions, and organizational size may further shape both experiential narratives and viable response pathways (19). Multilevel prevention strategies should therefore be adapted to local conditions when applied elsewhere.

## 5 Limitations

Several limitations warrant consideration. First, online Q&A data capture individuals who actively seek information and share experiences in digital spaces and thus may not represent the broader workforce; accordingly, the corpus should be interpreted as an auxiliary signal rather than a population estimate. This participation structure may overrepresent workers who are digitally literate, comfortable with anonymous online disclosure, or actively seeking advice about formal or procedural responses, while underrepresenting those who do not use digital help-seeking channels or whose experiences remain unposted. Second, because the corpus was assembled using the keyword “workplace bullying,” analyses are inherently bounded to narratives in which users explicitly adopt this label; relevant experiences described using alternative labels (e.g., “power abuse,” “conflict,” or “stress”) may be underrepresented. Third, cross-period comparisons are descriptive. Topic models were fitted separately by period and thematic frames were compared; however statistical tests of changes in topic prevalence were not conducted, nor were causal effects of legal reform estimated. Fourth, noun-centered tokenization—common in Korean topic modeling—may omit evaluative content conveyed through verbs, adjectives, and negation, potentially underrepresenting stance-taking or moral appraisal. Fifth, topic modeling captures corpus-level co-occurrence patterns and does not recover within-case temporal sequencing; topic labeling also necessarily involves interpretive judgment. Accordingly, any temporal interpretation in this study should be understood as a comparison of corpus-level thematic organization between document collections, not as evidence of longitudinal change within individual cases. Although topic interpretation was informed by iterative inspection of top terms and representative documents, transparency could be further strengthened by specifying labeling rules, inter-rater agreement procedures, and sensitivity checks more explicitly. Finally, discursive patterns were not linked to external outcomes (e.g., formal reporting, service utilization, sickness absence, turnover), limiting inference regarding behavioral or health impacts.

### 5.1 Future research directions

Future work could strengthen inference about temporal change by incorporating document-level metadata (e.g., year, sector, employment type) and modeling topic prevalence as a function of covariates using Structural Topic Models [STM; (84)], or by modeling temporal dynamics directly via Dynamic Topic Models [DTM; (85)]. Robustness could also be enhanced through systematic sensitivity analyses (e.g., alternative preprocessing choices, tokenization schemes, and stability checks across random seeds) and triangulation with qualitative methods. Substantively, priority directions include (1) linking discursive reorganization to downstream outcomes (e.g., reporting behavior, administrative or legal pathway use, mental health service utilization, sickness absence, turnover), (2) identifying organizational conditions that facilitate a shift from “knowing the law” to “using the law safely,” (3) addressing potential underrepresentation of vulnerable groups through targeted sampling or complementary data sources, and (4) evaluating multilevel prevention strategies using explicit implementation and process measures aligned with public health frameworks. In particular, future studies should consider strategies to capture the experiences of workers who may be underrepresented in mainstream online forums. For example, researchers could collect data from specialized communities frequented by certain vulnerable groups (e.g., anonymous nurse forums, call-center worker online communities, or migrant worker support websites) to ensure a more inclusive sample of bullying narratives. Qualitative approaches, such as in-depth interviews or focus groups with contract workers or foreign employees, could complement online text analysis to bring forward voices that might otherwise be overlooked. Indeed, recent surveys show that non-regular (freelance, temporary, or subcontracted) workers report higher rates of bullying than regular employees in Korea (86), underscoring the importance of including such groups in research. To achieve this, multi-language data collection and translation may be necessary; for instance, incorporating content from migrant workers' social media in their native languages could broaden the cultural scope of discourse analysis. By expanding data sources and methods, future research can better address the blind spots of this study and deepen our understanding of how workplace bullying is discussed and managed across diverse populations.

## 6 Conclusion

Workplace bullying constitutes a structural public health concern that requires coordinated multilevel prevention and response. Drawing on naturally occurring online help-seeking narratives accumulated over 6 years following legal regulation in South Korea, we observed corpus-level thematic patterns suggesting a more procedurally framed bullying discourse in the later-period corpus, with greater visibility of documentation, investigation, and institutional remedy pathways, while expressions of distress remained prominent. These findings should be interpreted as descriptive contrasts between corpora rather than as causal evidence of policy-driven change. This more procedurally framed pattern may be double-edged: without implementation designs that prioritize safety, protection, and recovery, formal systems may inadvertently intensify evidentiary burdens and risks of secondary victimization. Integrating primary prevention (organizational design and leadership), secondary prevention (safe reporting and high-quality investigations), and tertiary prevention (treatment and employment recovery support) into a coherent continuum remains central to reducing population-level harms.

## Data Availability

The data analyzed in this study is subject to the following licenses/restrictions: The data analyzed in this study were collected from publicly accessible posts on Naver Knowledge iN. Due to platform terms of service and privacy considerations, the compiled dataset cannot be publicly shared. Processed data and analysis code are available from the corresponding author upon reasonable request. Requests to access these datasets should be directed to Dong-gwi Lee, lee82@yonsei.ac.kr.
