# Supplementary material for: Topic modeling of workplace bullying discourse following legal regulation in South Korea
Source: Front Public Health. 2026 May 5;14:1811545. doi: 10.3389/fpubh.2026.1811545 (PMC13183848; doi:10.3389/fpubh.2026.1811545)
Supplement: Supplementary file 1 [file Data_Sheet_1.docx]

Supplementary Methods

# Text Preprocessing and Tokenization Details

This section documents the preprocessing resources and rules used in the LDA workflow. The description is based on the rule-based preprocessing pipeline and the domain-specific lexicon used in the analysis. The aim is to provide sufficient detail for readers to evaluate preprocessing decisions and facilitate reproducibility of the analytic workflow, with key rules and examples presented for transparency.

# Domain-specific Lexicon

The lexicon was constructed through a hybrid procedure combining corpus-driven frequency analysis and manual expert review. First, a full frequency list of candidate tokens was generated from the corpus. Two researchers independently reviewed high-frequency tokens and proposed classifications (e.g., stopword vs. content word) as well as normalization rules. Disagreements were resolved through discussion until consensus was reached. This procedure was intended to reduce individual bias and enhance the consistency of preprocessing decisions.

The resulting lexicon was used to support token filtering and normalization. It contained 16,611 entries and included the following fields used in preprocessing: token, frequency (freq), part-of-speech tag (pos), category, and normalized form (when available). The category field was used to distinguish ordinary lexical items from stopwords, whereas the normalized-form field was used to collapse lexical variants or multiword expressions into a unified representation.

Supplementary Table S1 presents representative examples of lexicon-based preprocessing rules applied in the study. The lexicon was developed through corpus-level frequency inspection and iterative refinement, and the examples shown here illustrate major types of filtering and normalization rules.

**Supplementary Table S1.** Representative examples of lexicon-based preprocessing rules.

| **Category** | **Original expression(s)** | **Standardized form** | **Rule type** | **Rationale** |
| --- | --- | --- | --- | --- |
| Workplace bullying | 가스, 라이팅 | 가스라이팅 | token merging | Frequently co-occurring tokens representing a unified concept |
| Hierarchical relations | 팀장, 상사, 관리자 | 상사 | synonym unification | Conceptually overlapping hierarchical roles |
| Workplace behavior | 무시하다, 무시 | 무시 | normalization | Verb–noun variation consolidated |
| Discourse marker | 그냥, 좀, 약간 | (removed as stopword) | stopword removal | High-frequency non-informative expressions |

# Stopword Resources

Stopwords were removed when they met one or more of the following criteria: (a) high-frequency but low semantic specificity, (b) functioning as grammatical or discourse markers, or (c) redundancy across nearly all documents. This approach is consistent with common practices in topic modeling, where non-informative high-frequency terms are removed to improve topic coherence.

Stopword removal was implemented at two levels. First, lexicon-based stopwords were removed whenever an entry was categorized as a stopword in the domain-specific lexicon. Second, the preprocessing pipeline applied an additional manually curated stopword list, as well as a set of topic-common words, to further improve topic distinctiveness.

The manual stopword list primarily targeted pronouns, deictic expressions, politeness formulas, and low-information discourse terms. The topic-common list removed high-frequency words that appeared across most workplace-bullying posts and therefore contributed little to topic differentiation. Detailed categories and examples of removed stopwords are presented in Supplementary Table S2.

**Supplementary Table S2.** Stopword categories and removal criteria.

| **Category** | **Example expressions** | **Examples with English glosses** | **Removal criterion** | **Rationale** |
| --- | --- | --- | --- | --- |
| Lexicon-based stopwords | 등등, 만약, 아니, 물론, 약간, 반면, 만일, 해당, 얘기, 얼마, 조금, 내공, 완전, 제목, 본인, 이야기 | etcetera; if; no/not; of course; somewhat/slightly; on the other hand; if (formal); relevant/corresponding; talk/story; how much/how many; a little; experience level/skill (online slang); completely/totally; title; oneself; story/talk | High-frequency terms identified during lexicon construction with low discriminative value | Frequently occurring terms that do not contribute to topic differentiation |
| Manual stopwords | 저, 제, 제가, 내, 너, 그, 이, 것, 요, 때, 후, 등, 수, 말, 일, 내, 본인, 자기, 쪽, 편, 분, 사람, 거, 안녕하세요, 안녕, 감사, 감사합니다, 부탁, 문의, 질문, 얘기, 이야기, 부분, 라고 | I (formal); my (formal); I (subject, formal); my (informal); you (informal); that; this; thing; polite ending marker; time/when; after; etcetera; way/possibility; speech/words; work/matter; oneself; oneself/self; side; side/tendency; person (honorific); person; thing (colloquial); hello (formal); hi; thanks; thank you (formal); request/favor; inquiry; question; talk/story; story/talk; part; quotative marker (“that…”) | Function-like or conversational terms identified during preprocessing | Non-content-bearing expressions reflecting conversational style rather than thematic content |
| Topic-common words | 직장, 회사, 괴롭힘, 직장내괴롭힘, 문제, 상황, 생각, 정도, 때문, 관련, 내용, 경우, 이유, 사실, 상태, 이상, 시간, 개월 | workplace; company; bullying; workplace bullying; problem; situation; thought; degree/extent; because of/due to; related/relation; content; case; reason; fact; state/condition; abnormality/more than (context-dependent); time; months | High-frequency topic-general terms appearing across most documents | Terms that are central but overly ubiquitous, reducing topic differentiation in LDA |

# Sensitivity Analysis

To evaluate the potential impact of preprocessing decisions, sensitivity analyses were conducted by comparing topic models with and without selected stopword and normalization rules. The overall topic structure and interpretability were consistent across model specifications, indicating that preprocessing choices did not artificially induce specific thematic patterns. Detailed quantitative results of these analyses, including coherence scores, perplexity, and vocabulary size across preprocessing conditions, are reported in Supplementary Table S5.

# Normalization Rules and Token-combination Rules

Normalization was applied conservatively and limited to cases where multiple surface forms clearly referred to a single conceptual entity. No semantic merging across distinct concepts was performed. These normalization and merging rules were applied conditionally when the tokens appeared together as parts of a single semantic expression.

Normalization was performed at both the text-cleaning stage and the token stage. Text-level normalization was applied before morphological analysis using rule-based substitutions. Token-level normalization was then applied using the normalized-form field in the lexicon.

After token extraction, additional compound-token merging rules were applied to preserve analytically central expressions as single units. Representative examples included: (a) 실업 + 급여 → 실업급여, (b) 권고 + 사직 → 권고사직, and (c) 직장 + 내 + 괴롭힘 → 직장내괴롭힘.

**Supplementary Table S3.** Text normalization and token merging rules applied in preprocessing

| **Rule type** | **Original form** | **Normalized output** | **Rationale** |
| --- | --- | --- | --- |
| Text-level replacement | 실업 급여 | 실업급여 (unemployment benefits) | Preserve a multiword policy/legal term as one unit |
| Text-level replacement | 권고 사직 | 권고사직 (recommended resignation / forced resignation) | Preserve a labor-related expression as one unit |
| Text-level replacement | 직장 내 괴롭힘 | 직장내괴롭힘 (workplace bullying) | Preserve the focal concept as one token |
| Lexicon normalization | 사생활 침해 | 사생활침해 (privacy invasion) | Standardize spacing for a conceptually unified term |

# Tokenization Procedure

Morphological analysis was conducted using Komoran, a widely used Korean morphological analyzer. The token-selection logic followed a lexicon-first decision rule. If a candidate token matched an entry in the lexicon, the script first checked whether it was a stopword; stopwords were removed. If a normalized form was available, the normalized form was used. If no stopword flag and no normalization rule applied, the token was retained only when it was tagged as a noun in the lexicon. For tokens not found in the lexicon, only Komoran tokens with noun tags (NN*) and a length of at least two characters were retained.

1. Basic text cleaning: strip whitespace, collapse repeated spaces, and remove repeated exclamation/question marks.
2. Rule-based text normalization for key multiword expressions before morphological analysis.
3. Sentence segmentation by line breaks and punctuation.
4. Morphological analysis with Komoran.
5. Lexicon-based filtering and normalization.
6. Retention of noun tokens only when no lexicon-based exclusion applied.
7. Post-tokenization compound merging for analytically central expressions.

These steps were applied sequentially in the order listed above.

# Note on Nouns-only Tokenization

The final topic-modeling corpus was constructed using a nouns-only tokenization rule. This choice is common in Korean topic-modeling studies because noun tokens tend to carry the most stable referential content and often improve topic interpretability. However, the approach may also discard evaluative or interactional information expressed through verbs, adjectives, and sentence endings. In the present study, this limitation was addressed at the interpretation stage by examining representative documents together with the top-ranked words for each topic rather than relying on keyword lists alone.

For transparency, representative examples of the lexicon and preprocessing rules are presented in the tables above.

# Reproducibility statement

To enhance transparency, this appendix reports the main preprocessing principles, stopword categories, normalization rules, and representative examples used in the analytic workflow. These descriptions are intended to allow readers to evaluate the rationale and scope of preprocessing decisions.
