# Supplementary material for: Topic modeling of workplace bullying discourse following legal regulation in South Korea
Source: Front Public Health. 2026 May 5;14:1811545. doi: 10.3389/fpubh.2026.1811545 (PMC13183848; doi:10.3389/fpubh.2026.1811545)
Supplement: Supplementary file 2 [file Data_Sheet_2.docx]

Supplementary Tables

# Supplementary Table S4. Representative examples of included and excluded posts following data filtering.

| **Category** | **Filtering criterion** | **Description** | **Example (translated)** |
| --- | --- | --- | --- |
| Included | Workplace bullying (power imbalance, repeated behavior) | Posts describing ongoing mistreatment within hierarchical relationships | “A physician insulted me in front of colleagues, causing ongoing fear and distress. Despite organizational response, the issue remains unresolved due to power imbalance.” |
| Included | Workplace bullying (repeated interpersonal conflict) | Conflicts involving colleagues or team dynamics within work context | “A senior colleague keeps pressuring me to stay late and finish tasks, even when they are not urgent, while he leaves work early himself. He often questions whether I can leave and tells me to stay until everything is done.” |
| Excluded | Sexual harassment (without broader bullying context) | Posts describing sexual harassment as the primary issue, without broader patterns of repeated workplace bullying | “I reported a workplace sexual harassment case, but the company refused to disclose the disciplinary outcome and declined to reopen the investigation despite additional incidents involving the same perpetrator.” |
| Excluded | One-time interpersonal conflict | Isolated incidents without repetition or structural imbalance | “I was physically assaulted by a colleague at a company dinner, but the incident was a one-time event and did not involve repeated or sustained mistreatment.” |
| Excluded | General inquiry / informational content | Questions or discussions not describing personal experience | “I found information online about workplace bullying regulations, but I am unsure how to revise company policies or report compliance to the Ministry of Employment and Labor.” |
| Excluded | Irrelevant content | Content unrelated to workplace bullying | “I work evening shifts and am unsure whether I am entitled to weekly holiday pay, night pay, and wages during the training period. How should my salary be calculated?” |

# Supplementary Table S5. Sensitivity analysis of preprocessing decisions in topic modeling

| **Model** | **Stopword Removal** | **Normalization** | **Coherence (c_v)** | **Log Perplexity** | **Vocabulary Size** |
| --- | --- | --- | --- | --- | --- |
| Baseline | Yes | Yes | 0.472 | -6.469 | 2,041 |
| No Stopword | No | Yes | 0.439 | -6.544 | 2,495 |
| No Normalization | Yes | No | 0.450 | -6.471 | 2,051 |

# Supplementary Table S6. Model evaluation metrics (c_v, c_npmi, perplexity) for LDA models with K = 2–18

| **K** | **c_v Coherence** | **c_npmi Coherence** | **Log Perplexity** |
| --- | --- | --- | --- |
| 2 | 0.3537 | 0.0005 | -6.9816 |
| 3 | 0.3719 | 0.0031 | -6.9617 |
| 4 | 0.3773 | 0.0057 | -6.9611 |
| 5 | 0.3667 | 0.0099 | -6.9646 |
| 6 | 0.3534 | 0.0054 | -6.9765 |
| 7 | 0.3576 | 0.0061 | -6.9939 |
| 8 | 0.3522 | 0.0032 | -6.9948 |
| 9 | 0.3513 | 0.0021 | -7.0113 |
| 10 | 0.3607 | 0.0037 | -7.0116 |
| 11 | 0.3654 | 0.0019 | -7.0207 |
| 12 | 0.3848 | 0.0111 | -7.0194 |
| 13 | 0.3826 | 0.0116 | -7.0274 |
| 14 | 0.3637 | 0.0062 | -7.0382 |
| 15 | 0.3570 | 0.0038 | -7.0457 |
| 16 | 0.3558 | 0.0042 | -7.0425 |
| 17 | 0.3659 | 0.0070 | -7.0488 |
| 18 | 0.3638 | 0.0022 | -7.0522 |

# Supplementary Table S7. Yearly prevalence (%) of topics identified by the 12-topic LDA model across 2019–2025. Values represent average document-level topic proportions.

| **year** | **2019** | **2020** | **2021** | **2022** | **2023** | **2024** | **2025** |
| --- | --- | --- | --- | --- | --- | --- | --- |
| **Topic_1** | 7.38% | 8.09% | 7.99% | 7.70% | 7.51% | 7.51% | 7.25% |
| **Topic_2** | 10.20% | 7.71% | 8.47% | 7.83% | 7.93% | 7.10% | 7.47% |
| **Topic_3** | 4.18% | 6.91% | 5.74% | 6.46% | 7.55% | 6.62% | 9.23% |
| **Topic_4** | 8.47% | 8.56% | 8.29% | 8.62% | 7.56% | 8.78% | 7.37% |
| **Topic_5** | 7.91% | 6.50% | 7.44% | 7.31% | 6.43% | 6.65% | 6.56% |
| **Topic_6** | 3.71% | 4.27% | 3.56% | 4.23% | 3.83% | 4.17% | 4.42% |
| **Topic_7** | 11.27% | 11.26% | 10.50% | 10.52% | 11.22% | 11.64% | 10.56% |
| **Topic_8** | 16.58% | 14.15% | 14.04% | 13.06% | 12.68% | 12.94% | 12.55% |
| **Topic_9** | 9.10% | 8.24% | 8.07% | 7.86% | 8.74% | 8.38% | 8.31% |
| **Topic_10** | 7.15% | 8.35% | 9.09% | 9.46% | 10.58% | 10.57% | 12.26% |
| **Topic_11** | 8.05% | 10.88% | 11.92% | 11.87% | 10.88% | 10.51% | 9.55% |
| **Topic_12** | 6.00% | 5.07% | 4.90% | 5.07% | 5.08% | 5.12% | 4.46% |
